# Localization of α 2u-globulin in the acinar cells of preputial gland, and confirmation of its binding with farnesol, a putative pheromone, in field rat (*Millardia meltada*)

**DOI:** 10.1371/journal.pone.0197287

**Published:** 2018-06-01

**Authors:** Ramachandran Rajamanickam, Achiraman Shanmugam, Rajagopal Thangavel, Sankarganesh Devaraj, Kamalakkannan Soundararajan, Ponmanickam Ponnirul, Rajkumar Ramalingam, Ramya Vaideki Ganesan, Padmanabhan Parasuraman, Archunan Govindaraju

**Affiliations:** 1 Department of Environmental Biotechnology, Bharathidasan University, Tiruchirappalli, Tamilnadu, India; 2 Centre for Pheromone Technology, Department of Animal Science, Bharathidasan University, Tiruchirappalli, Tamilnadu, India; 3 Post Graduate and Research Department of Zoology and Microbiology, Thiagarajar College (Autonomous), Madurai, Tamilnadu, India; 4 Department of Zoology, Bishop Heber College (Autonomous), Tiruchirappalli, Tamilnadu, India; 5 Department of Zoology, Ayya Nadar Janaki Ammal College (Autonomous), Sivakasi, Tamilnadu, India; 6 Nuclear Dynamics and Architecture Lab, Institute of Medical Biology-IMB, Singapore, Singapore; 7 Translational Neuroscience Laboratory, Lee Kong Chian School of Medicine, Nanyang Technological University, Singapore, Singapore; Centre for Cellular and Molecular Biology, INDIA

## Abstract

Pheromones, low molecular weight chemical entities that bind to pheromone carrier proteins, are chemical signals that play an important role in the communication system in animals. This has been rather fairly well-studied in the rodents. The preputial gland, a rich source of pheromones in many rodents, contains a low molecular mass protein (18–20 kDa) that acts as one such pheromone carrier. However, the presence of this protein in the notorious rodent pest *Millardia meltada* has not yet been proven. Therefore, we aimed at identifying this protein, and the pheromones that are bound to it, in this rodent so as to utilize the information in the control of this pest. Twenty volatile compounds were identified in the preputial gland using GC-MS. Total protein of the gland was fractioned by both one and two-dimensional electrophoresis when we identified a low molecular mass protein (19 kDa, pI-4.7). Adopting MALDI-TOF MS and LC-MS analyses, the protein was confirmed as α 2u-globulin. To identify the volatiles bound to this protein, we used column chromatography and GC-MS. We found that farnesol and 6-methyl-1-heptanol are the volatiles that would bind to the protein, which we propose to be putative pheromones. Immunohistochemical analysis confirmed localization of α 2u-globulin in the acinar cells of the preputial gland. Thus, we show that α 2u-globulin, a pheromone-carrier protein, is present in the preputial gland acinar cells of *M*. *meltada* and suggest farnesol and 6-methyl-1-heptanol to be the volatiles which would bind to it. The α 2u-globulin together with farnesol and 6-methyl-1-heptanol contribute to pheromonal communication of *M*. *meltada*.

## Introduction

Pheromones play inevitable roles in mammalian reproduction and social behavior including sexual attraction [1], territorial marking [2], mother-young interactions [3], conspecifics identification [4] and aggression [5]. The major sources of pheromones are urine, feces, saliva, vaginal mucus, sweat, scent glands, etc. [6, 7]. In the rodents the preputial gland, a modified sebaceous gland, located subcutaneously on both sides of the midline just superior to the symphysis pubis, is sexually dimorphic and bilaterally symmetrical [8]. The cells of the male preputial gland undergo hypertrophy, with accumulation of cytoplasmic lipid droplets, at a time close to sexual maturation. The gland also contains sex pheromones in it [8, 9].

The pheromone carriers are important components in mobilizing the pheromones. They are mostly proteins with a low molecular mass [10, 11]. These barrel-shaped (eight-stranded β-barrel) protein molecules belong to the superfamily of lipocalins and possess a hydrophobic cavity that serves as a “container” for the volatile ligands [12]. This cavity binds small hydrophobic molecules including a wide range of odorants [13]. These proteins are involved both in the perception (odorant binding proteins) as well as delivery of chemical signals (pheromone carrier proteins) in various animal species [14, 15]. The proteins were reported in mouse and rat urine [16, 17], hamster vaginal secretion [18], pig saliva [19], human sweat [20], horse sweat [21], and buffalo saliva [22]. The pheromone-protein complex slowly releases the odorants [17] and is crucial in protecting the pheromones from rapid evaporation, eventually extending the shelf-life of the scent mark [23]. In addition, these proteins resist high temperature and are not likely to be fast denatured when released into the environment.

In our earlier studies, we identified volatiles bound with α 2u-globulin in the preputial gland [24] and urine [25] of house rat. The pheromonally active compounds in rodents are volatile in nature and tend to bind to the proteins, and be excreted in urine. It has been suggested that the contents of preputial gland and the bladder urine are the major source of pheromone activity [26]. In the male field rat, the preputial gland is situated on each side of the penis and its main excurrent duct runs along the lateral surface and empties on the side of the urethral meatus, but not connected to the terminal urethra [8]. The above reports led to a query whether the carrier protein binds with the volatiles in the preputial gland and is then released into the urine or the volatiles from preputial gland are released into the urine first and then bind with the carrier protein. To clarify this point, we aimed at localizing the carrier protein and identification of the bound volatiles in the preputial gland during sebum formation in the field rat, *Millardia meltada*. Hitherto, neither the pheromones nor the carrier proteins have been reported in this rat. Therefore, the present study was undertaken to i) investigate the histomorphology and histochemistry of preputial gland of *M*. *meltada* to obtain direct evidence for the presence of proteins and lipids, ii) identify the volatiles, bound volatiles and carrier protein(s), iii) confirm the carrier protein(s) by MALDI-TOF MS and LC-MS analyses, and iv) localize the carrier protein by immunohistochemistry.

## Materials and methods

### Ethics statement

The experiments were approved and carried out in accordance with the Institutional Animal Ethics Committee (IAEC) of Bharathidasan University, India (Approval No. BDU/IAEC/2012/71).

### Experimental animals

Adult male field rats (*Millardia meltada*) were captured using traps in paddy fields near Bharathidasan University, Tiruchirappalli, India, under permission from land owners, transferred to the animal house and housed individually in polypropylene cages. They were fed pellet food (Sai Durga Feeds, Bangalore, India) and water *ad libitum*.

### Dissection of preputial gland

The preputial glands situated close to prepuce were traced following cervical dislocation [27]. A portion of the gland was dissected out and used for histological and immunohistochemical analyses, while another segment was used for extraction of volatiles, proteomic analysis and size-exclusion chromatography.

### Extraction of volatiles

Using a clean mortar and pestle, the gland was homogenized in dichloromethane. The extract was filtered, and analyzed by Gas Chromatograph-Mass Spectrometer (GC-MS) for identification of volatiles.

### Histomorphological and histochemical analyses

The tissue was fixed in neutral buffered formalin and processed for embedding in paraffin wax. A rotary microtome was used to obtain sections at 4–6 μm thick paraffin sections that were mounted to glass slides, stained with hematoxylin and eosin, dehydrated in alcohol, cleared in xylene and mounted in DPX resin. A light microscope was used to view and describe the various cell types in the gland. Sections were also stained with Sudan Black B and Fast Green FCF for histochemical analysis.

### Gas Chromatography-Mass Spectrometry (GC-MS) analysis

The GC-MS analysis was performed by injecting 2 μL of solvent extract into the injector port of the GC-MS (QP-5000, Shimadzu, Japan). A 30-m glass capillary column with film thickness of 0.25 μm (30 m x 0.2 mm i.d., coated with UCON HB 2000) was fitted in the GC-MS. The oven temperature was initially 40°C for 4 min, increased to 250°C 10 min^-1^ and hold at 250°C for 11 min. The mass spectrometer was used in electron ionization mode (70 eV) and helium was used as reagent gas (1.2 mL/min). The resulting mass spectra of individual unknown compounds were compared with the library (Wiley and NIST) and identified by probability-based matching.

### Purification of α 2u-globulin by column chromatography and identification of protein-bound volatiles

The preputial gland homogenized with phosphate buffered saline (PBS) was loaded on the top of the gel filtration column, which was packed with Sephadex G-50 dissolved in 10mM Tris-HCl (pH 7.2). The fractions were eluted, collected (1.5 mL/fraction) and analysed by Bradford method for the presence of protein. Appropriate fractions that contained low molecular weight proteins were separated using sodium dodecyl sulfate-polyacrylamide gel electrophoresis (SDS-PAGE) [28]. The eluted fractions that contained the protein of interest (19 kDa) were pooled and extracted with dichloromethane, and subsequently analysed in GC-MS to identify the protein-bound volatiles [24].

### Two-dimensional gel electrophoresis

The two-dimensional (2D) gel electrophoresis was carried out adopting the protocol of Rajkumar et al. [29]. Analytical isoelectric focusing was performed under native condition using 5% polyacrylamide gel in a gradient of ampholytes (pH 3.5 to 9.5).

### Destaining and in-gel digestion

Protein bands from SDS-PAGE were excised and each gel plug was destained using 100 mL of 25 mM ammonium bicarbonate and 50% (v/v) acetonitrile (1:1), and incubated at 37°C for 30 minutes. This step was repeated until no stain was visible in the gel band. The protein band was sliced into small cubes and placed in 1.5-mL Eppendorf tubes. After drying in a Speed-Vac (Savant), the cubes were incubated in 100 μL of 2% β-mercaptoethanol/25mM NH_4_HCO_3_ in the dark for 20 min at 25°C. The same volume of 10% 4-vinyl pyridine in 25 mM NH_4_HCO_3_/50% acetonitrile was added for cysteine alkylation. After a 20-min incubation, the gel was soaked in 1 mL of 25 mM NH_4_HCO_3_ for 10 min, dried and then incubated with 25 mM NH_4_HCO_3_ containing 100 ng of modified trypsin (Promega) overnight (~18 h). The tryptic digests were removed from the gel, extracted with 300 mL of 25 mM NH_4_HCO_3_ and 50% acetonitrile, respectively. These two fractions were pooled, dried in a Speed-Vac and the digested peptide was re-suspended in 0.1% formic acid and analysed in MALDI-TOF MS [24].

### Matrix-assisted laser desorption/ionization-mass spectrometry analysis

The tryptic digests were prepared by mixing equal amounts (2:2) of peptide mixture with the matrix solution (α-cyano-4-hydroxycinnamic acid) saturated with 0.1% trifluoroacetic acid and acetonitrile (1:1). Then the samples were analyzed in reflectron mode with a delay time of 90 ns and 25 Kv accelerating voltage in the positive ion mode. To improve the signal-to-noise ratio, the summation of 300 laser shots was taken for each spectrum. External calibration was done using peptide I calibration standard with masses ranging from 1046 to 3147 Da. Mass spectra were acquired using ULTRA FLEX-TOF/TOF mass spectrometer (Bruker Daltonics, Germany) equipped with a 337 nm pulsed nitrogen laser. MS-MS spectra were acquired by selecting the precursor mass with 8 Da window [24].

### Mascot data search analysis

Spectra were processed using flexAnalysis software. Monoisotopic peptide masses were assigned and used in the database search. The protein identification was accomplished utilizing the MASCOT database search engine (Matrix Science, London, UK) (http://www.matrixscience.com). Probability-based MW search scores were estimated by comparison of search results against an estimated random match population and were reported as 10 log10 (P), where P is the absolute probability. Scores >63 were shown to be significant (*P<0*.*05*) in the Mascot search. Proteins identified with scores less than the significant level were reported as unidentified.

### Liquid Chromatography-Mass Spectrometry Analysis (LC-MS)

The protein of interest was cut out from the SDS-PAGE and subjected to trypsin digestion, and then the LC-MS analysis was carried out [24]. Mass spectrometric analyses were performed using an LTQ-Orbitrap (Discovery) hybrid mass spectrometer with a nanoelectrospray ionisation source (Thermo Electron, San Jose, CA, USA) coupled to a nano-flow high-performance liquid chromatography (HPLC) system (Agilent Technologies 1200 series, Germany). An Agilent C18 column (100 × 0.075 mm, 3.5 mm particle diameter) with mobile phases of 0.1% formic acid in water and in acetonitrile were used. The pump flow rate was 0.5 mL/min, and peptide elution was achieved using a linear gradient of 5%–35% B for the first 30 min followed by a rapid increase to 95% B over the next 10 min. The conventional MS spectra (Survey Scan) were acquired at high resolution (M/DM, 60,000 full widths half maximum) over the acquisition range of *m/z* 200–2000 and a series of precursor ions were selected for the MS/MS scan.

### Immunohistochemical analysis of preputial gland

The sections mounted onto glass slides were deparaffinised and brought down to water followed by quenching the endogenous peroxidase activity. Primary antibody (Rat α 2u-globulin-specific polyclonal goat IgG) was applied to the slide initially, and rinsed with buffer followed by the addition of secondary antibody (HRP-conjugated Rabbit Anti-Goat IgG). The sections were rinsed with buffer, the detection complex (DAB chromogen) was added and the sections were observed in a light microscope and recorded.

## Results

### Histomorphology and histochemistry of preputial gland

**Fig 1:**
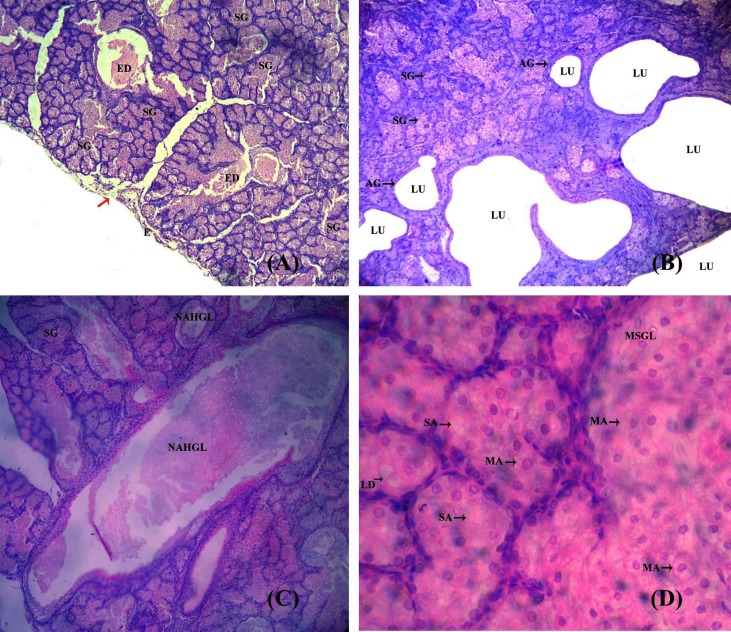
**(A-D)**: Histological structure of preputial gland of *Millardia meltada***: ED**-Excretory duct; **E**-Epidermis; **SG**-Sebaceous gland; **Red arrow-**the branched large sebaceous glandular lobules directly open into the skin surface; **AG**-Apocrine gland is a simple tubular gland and the secretion are found in the lumen (**LU**); **NAHGL**-Normal alveolar holocrine glandular lobules; **MSGL**-Modified sebaceous glandular lobules; **SA**-Serous acini; **MA**-Mucus acini; **LD**-Lipid droplets.

**Fig 2:**
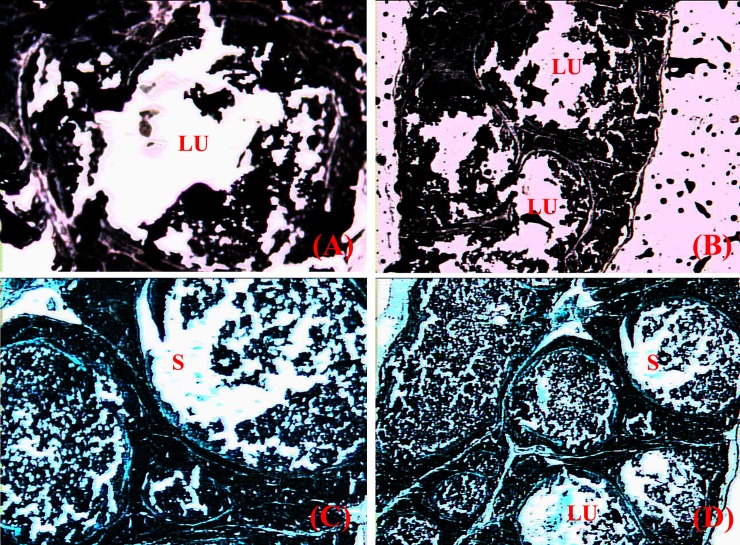
**(A-D)**: Histochemical features of preputial gland of *Millardia meltada*: **S**-sebum, **LU**-lumen.

The preputial glands are a pair of oval-shaped glands located as one on each side of the prepuce just beneath the epidermis. The gland is surrounded by richly vascularised connective tissue (**Fig 1**). It is a modified sebaceous gland, a simple tubular apocrine gland. The gland is protruded into lobes of various sizes, each containing a secretion-filled lumen, and each in a different state of activity. Microscopic observation revealed the presence of parenchymatous acini. The developing acini lack lumen and are formed of cells containing little cytoplasm and small nuclei. There are a few clear enlarged acini are containing sebum (**Fig 1**). Histochemical analysis with Sudan Black B evidenced fatty substances in the sebum (**Fig 2A and 2B**) and Fast Green FCF staining evidenced proteins in the sebum (**Fig 2C and 2D**).

### Volatile profiling in the preputial gland extract

**Fig 3:**
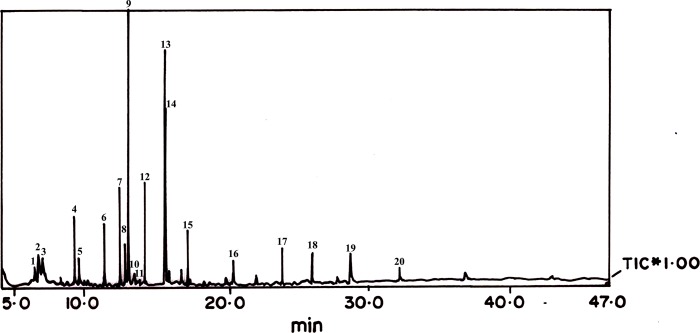
GC-MS chromatogram of crude extract of preputial gland of *Millardia meltada*. The fractions are Undecane, Dodecane, Tridecane, Decane, Tetradecane, 1-chloro-octadecane, 6-methyl-1-heptanol, (ethenyloxy)-isooctane, 1,3-bis(1,1-dimethylethyl)-benzene, Pentadecane, 1-(3,4-dimethylphenyl)-ethanone, Heptadecane, Farnesol, 4,6-dimethyl dodecane, 1,1’-oxybis-octane, 1-dodecanol, 3,4-dimethyl-heptane, Pentadecane, 3,5-bis(1,1-dimethylethyl)-phenol, and 1-iodo-decane.

The GC-MS analysis revealed twenty detectable peaks most of which were alkanes, phenols, alcohols, ketones and alkenes, with molecular weight between 128 and 268 Da. There were six major peaks, identified as 1-chloro-octadecane, 6 methyl-1-heptanol, farnesol, 1-dodecanol, pentadecane and 3,5-bis(1,1-dimethylethyl)-phenol, respectively (**Table 1; Fig 3**).

**Table 1:** List of compounds identified in the crude extract of preputial gland of *Millardia meltada*.

Peak No	Molecular Weight	Molecular Formula	Compound Name
1	156	C_11_H_24_	Undecane
2	170	C_12_H_26_	Dodecane
3	184	C_13_H_28_	Tridecane
4	142	C_10_H_22_	Decane
5	184	C_13_H_28_	Tetradecane
6	254	C_18_H_38_	1-chloro-octadecane
7	130	C_8_H_18_O	6-methyl-1-heptanol
8	156	C_10_H_20_O	(ethenyloxy)-isooctane
9	190	C_14_H_22_	1,3-bis(1,1-dimethylethyl)-benzene
10	156	C_11_H_24_	Pentadecane
11	148	C_10_H_12_O	1-(3,4-dimethylphenyl)-ethanone
12	198	C_14_H_30_	Heptadecane
13	222	C_15_H_26_O	Farnesol
14	226	C_16_H_34_	4,6-dimethyl dodecane
15	242	C_16_H_34_O	1,1’-oxybis-octane
16	186	C_12_H_26_O	1-dodecanol
17	128	C_9_H_20_	3,4-dimethyl-heptane
18	212	C_15_H_32_	Pentadecane
19	206	C_14_H_22_O	3,5-bis(1,1-dimethylethyl)-phenol
20	268	C_10_H_21_I	1-iodo-decane

### Proteomic profiling in the preputial gland extract

**Fig 4:**
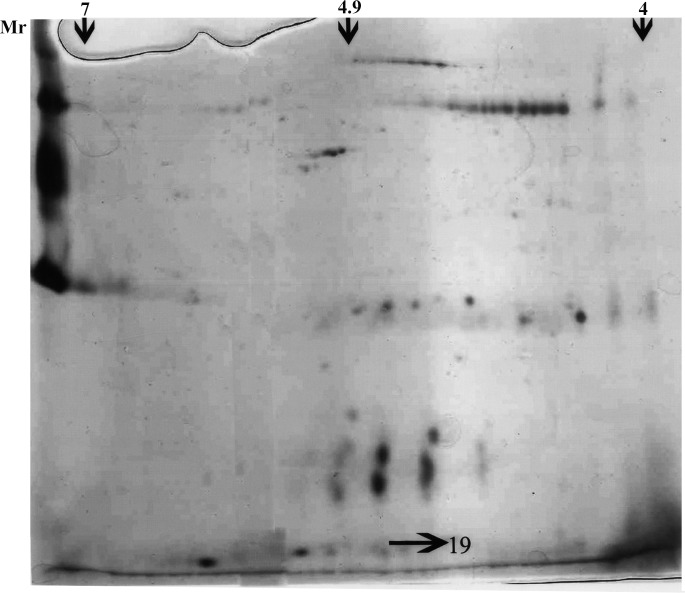
Protein profile in the representative 2-Dimensional electrophoresis of protein extract of preputial gland of *Millardia meltada*.

**Fig 5:**
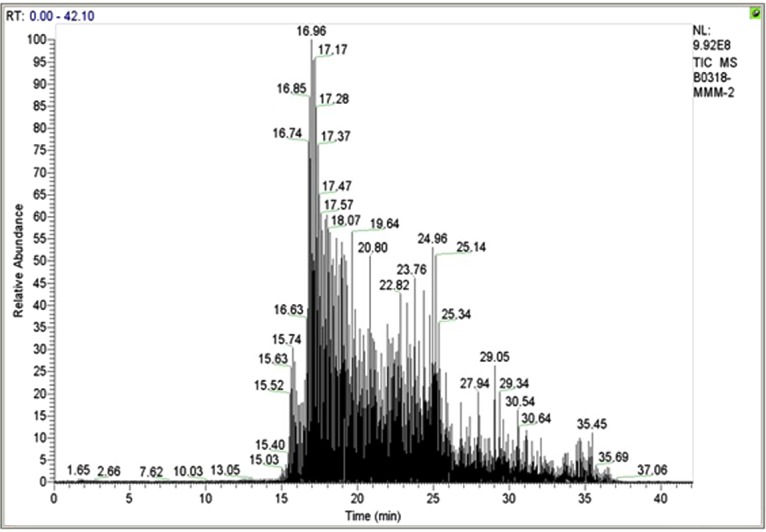
Mass spectrum by LC-MS.

Two-dimensional gel electrophoresis of the glandular extract revealed proteins with pI values in the range from 4 to 7. The 19 kDa candidate protein was of pI value 4.9 (**Fig 4**). MALDI-TOF MS fragmentation analysis for additional structural information revealed a sequence of eleven amino acids (DNIIDLTKTDR). When this was subjected to BLAST search to find the nature of the protein, the first five scores were 198, 174, 126, 93 and 68 indicating that the protein belongs to lipocalin family. The amino acid sequence corresponded to the α 2u-globulin of rat and the major urinary protein (MUP) of mouse. LC-MS analysis of the 19 kDa protein confirmed it to match the α 2u-globulin of *Rattus norvegicus* (**Fig 5**). The matched sequences are underlined and marked in bold.

**J** uptr:q63213_rat 4spectra zTot:0.0e0

>UPTR:Q63213_RAT Q63213 *Rattus norvegicus* (rat). Alpha 2u-globulin. 2/2005

MKLLLLLLCL GLTLVCGHAE EASFERGNLD VDKLNGDWFS IVVASDKREK IEENGSMR**VF VQHIDVLENS LGFTFR**IKENGVCTEFSLVA DKTAK**DGEYF VEYDGENTFT ILKTDYDNYV MFHLVNVNNG ETFQLMELYG R**TKDLSSDIK EKFAKLCVAHGITRDNIIDL TKTDRCLQAR G

**K** uptr:q9jji0_rat 3spectra zTot:0.0e0

>UPTR:Q9JJI0_RAT Q9jji0 *Rattus norvegicus* (rat). Alpha 2u-globulin. 6/2003

MKLLLLLLCL GLTLVCGHAE EASFKRGNLD VDKLNGDWFS IVMASDKREK IEENGSMRVF MQHIDVLENS LGFKFCIKVNGECRELYLVA YKTPK**EGEYF VEYDGGNTFN ILK**TDYDRYV MFHLVNFK**DG ETFQLMELYG R**TKDLSSDIK EKFAKLCVAHGITRENIIDV TKTDRCLQAR G

### Purification of α 2u-globulin by gel filtration chromatography

**Fig 6:**
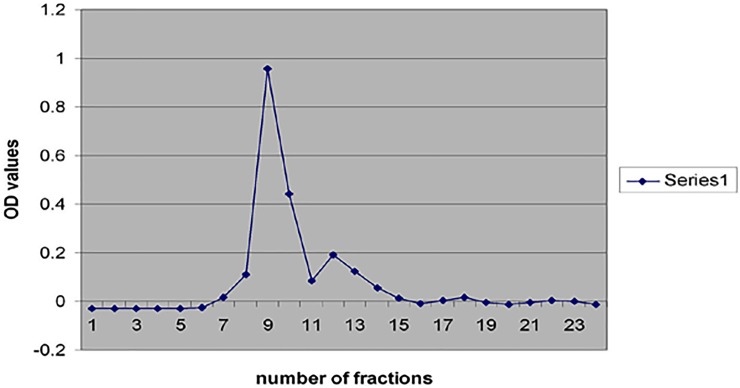
Detection of protein concentration in purified fraction collection.

**Fig 7:**
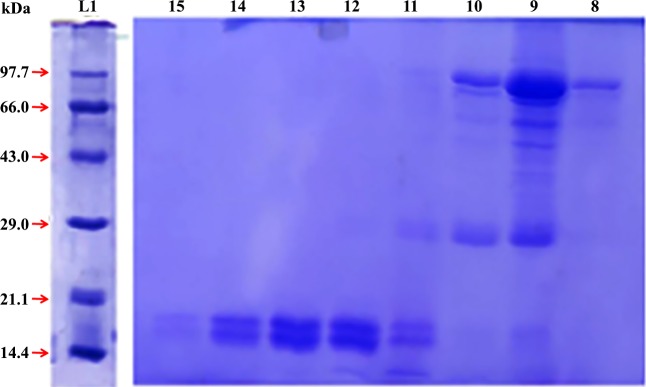
SDS-PAGE of purified protein fraction. Lane 1: Marker; Lanes 2–9: Purified protein (19kDa) from the crude extract.

There were 25 fractions to elute. They were collected and assessed for the presence of proteins (**Fig 6**). Eight fractions (8^th^ to 15^th^) were subjected to SDS-PAGE (**Fig 7)**. The 19 kDa protein was prominently expressed in fractions 12, 13 & 14. These fractions were pooled and used in GC-MS to identify protein-bound volatiles.

### Identification of bound ligands in the purified fractions

**Fig 8:**
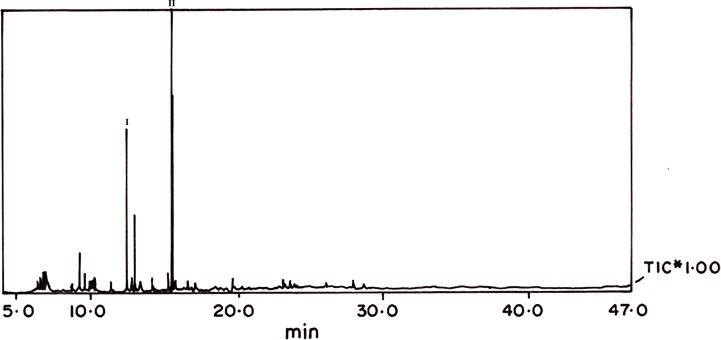
GC-MS identification of bound ligands as in the purified fractions 6-methyl-1-heptanol, and farnesol.

The pooled low molecular weight protein fractions showed two prominent peaks in GC-MS. The two peaks expressed characteristic matching ions with 6-methyl-1-heptanol and farnesol, respectively, in NIST-based library (**Table 2**; **Fig 8**).

**Table 2:** Bound form of volatiles in the purified fraction of preputial gland extract of *Millardia meltada*.

Peak No	Molecular Weight	Molecular Formula	Compound Name
I	130	C_8_H_18_O	6-methyl-1-heptanol
II	222	C_15_H_26_O	Farnesol

### Immunohistochemical localization of α 2u-globulin in the preputial gland

**Fig 9:**
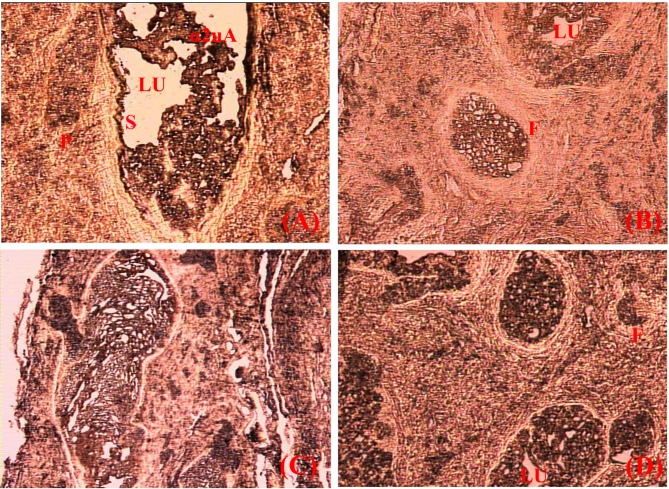
**(A-D)**: Immunohistochemical features of preputial gland of *Millardia meltada* **P**-primordial acini, **S**-sebum, **F**-fat substances, **LU**-lumen, **α2uA**-Alpha 2u-globulin antibody.

The reaction complex formed in the immunohistochemical analysis confirmed the presence of α 2u-globulin in the sebum of the gland (**Fig 9**). The cytoplasm of cells in well-developed acini showed the presence of α 2u-globulin. The hypertrophied cells were evidenced to release the sebum into the lumen (LU), which contained α 2u-globulin as revealed in the dark brown reaction product in the lumen of enlarged acini (**Fig 9**).

## Discussion

The preputial gland of rats is a specialised sebaceous gland that secretes sebum. The sebum is believed to contain pheromones. The major difference between a specialised and an ordinary sebaceous gland is the presence of different cell types with continuous formation of small to large lipid droplets therein in the former [30, 8]. We observed modified sebaceous gland with two cells types namely squamous cell (with single peripheral boundary) and acinar cell (with stratified epithelium containing a fatty substance in the cytoplasm). Histochemically, the enlarged acinar cells, with excess-stored fatty substances, were hypertrophied, disintegrated and fused with adjacent cells to form small to large lobules and the lumen was filled with sebum (a fatty substance). Atoji et al. [31] suggested four types of cells viz., peripheral, differentiating, mature and necrotic cells in the modified sebaceous glands. We found the cells adjacent to the core of the alveoli of the sebaceous gland to be filled with fat residues. The preorbital gland of dominant male blackbuck has both sebaceous and apocrine secretory glands [32]. The modified sebaceous glands in the scent glands of several antelopes have been reported [33–37].

The scent glands are often modified sebaceous glands and produce pheromones. These pheromones play crucial roles in conveying information about species, sex, and dominance status [38–41]. They are also sex attractants [7]. During scent marking, the scent produced by the sebaceous gland is adhesive and lasts longer [30, 32, 42]. Also, in rodents there are possibilities that the secretions can be smeared on the path the animal travels to serve as scent marks for their conspecifics to recognise [43]. Putting this information together we speculate that the lipid-rich material in the sebum may be involved in adhesion of the scent material with stones and pebbles when rats are on the move, which can be perceived by their conspecifics. These secretions also involved in territorial marking and social and reproductive behaviors [20]. The glands located adjacent to the genitalia help in reproductive behavior, whereas compounds from glands located on ventral, dorsal, head and flank regions are useful in marking territory and in other social behaviors [44–45].

The volatile compounds present in the preputial gland and urine of house mouse and rat were identified, wherein their individuality was revealed [46, 41]. Farnesol is found in the preputial gland of field-, house- and laboratory rats [8, 25]. Therefore, we suggest farnesol to be a pheromone of maleness in rats and be utilised as a candidate attractant. Together with other trace level compounds it may designate the species. We, in this study, identified 6 methyl-1-heptanol as a co-molecule with farnesol that identifies the maleness of the field rat. Preputial gland is anatomically situated adjacent to the penis and its main excurrent duct opens at the urethral meatus, but it is not connected to the terminal urethra [8]. Farnesol is present in urine as well as preputial gland of rats raises the question of farnesol’s ultimate origin. We have reason to claim that farnesol is produced by the preputial gland and released along with its other secretions into the urine.

The volatiles require efficient carrier molecule that may play crucial roles. Bacchini et al. [47] identified proteins as carriers of volatile molecules in mouse urine. Concurrently, protein carrier molecules were identified in mouse and rat [16, 17], hamster [18], pig [19], horse [21] and human [20]. These proteins were characterised as major urinary proteins (MUPs, 19 kDa) in mouse; α 2u-globulin (18 kDa) in preputial gland and urine of rat [24, 48]; aphrodisin (17 kDa) in hamster vaginal mucus [49]; salivary lipocalin (20 kDa) in boar [19]; and odorant binding protein in voles (OBP) [50] and rabbit [51]. Herein we identified a 19 kDa protein in the preputial gland of *Millardia meltada*. Size exclusion chromatography that was performed previously identified bound form of two volatiles 2-sec-butyl-4,5-dihydrothiazole and dehydro-brevicomin, in mice [23]. Armstrong et al. [52] also reported 2-sec-butyl-4,5-dihydrothiazole, as a protein-bound volatile in male mouse. Rajkumar et al. [25] identified the bound form of volatiles in house rat urine as 1-chlorodecane, hexadecane, 2,6,11-trimethyl dodecane and 2-methyl-N-phenyl-1,2-propenamide. We extracted specific fractions from the column and identified 6-methyl-1-heptanol and farnesol as protein-bound volatiles in *M*. *meltada*, which we suggest as the putative pheromones bound to the α 2u-globulin.

Lastly, we localized the carrier protein (α 2u-globulin) in the preputial gland in the immunohistochemical analysis. The histological sections of the preputial gland revealed the presence of acinar cells, sebum with fatty substances. Further, the immunohistochemical analysis with α 2u-globulin antibody proved the presence of α 2u-globulin in the sebum. The sebum released into the central duct of the preputial gland and possibly excreted through the urethra. Therefore, it is possible that sebum is excreted through urine and it is apparent that urine contains the volatile compounds farnesol and 6-methyl-1-heptanol, and their carrier protein, α 2u-globulin.

## References

[1] Achiraman S, Archunan G (2002) Urinary proteins and pheromonal communication in mammals. Indian J Exp Biol 40: 1077–1078 12587742

[2] Burger BV, Viviers MZ, Bekker JPI, Le Roux M, Fish N, Fourie WB, et al (2008) Chemical characterization of territorial marking fluid of male Bengal tiger, *Panthera tigris*. J Chem Ecol 34: 659–671 doi: 10.1007/s10886-008-9462-y 18437496 10.1007/s10886-008-9462-y

[3] Levy F, Keller M (2009) Olfactory mediation of maternal behavior in selected mammalian species. Behav Brain Res 200: 336–345 doi: 10.1016/j.bbr.2008.12.017 19146885 10.1016/j.bbr.2008.12.017

[4] Poddar-Sarkar M (1996) The fixative lipid of tiger pheromone. J Lipid Mediat Cell Signal 15: 89–101 9029376 10.1016/s0929-7855(96)00547-0

[5] Dominic CJ (1991) Chemical communication in animals (Banaras Hindu University). J Sci Res 41: 157–169

[6] Brennan PA, Kendrick KM (2006) Mammalian social odours: attraction and individual recognition. Philos Trans R Soc B Biol Sci 361: 2061–2078 10.1098/rstb.2006.1931 PMC1764843 17118924

[7] Archunan G (2009) Vertebrate pheromones and their biological importance. J Exp Zool India 12: 227–239

[8] Ponmanickam P, Palanivelu K, Govindaraj S, Baburajendran R, Habara Y, Archunan G (2010) Identification of testosterone-dependent volatile compounds and proteins in the preputial gland of rat *Rattus norvegicus*. Gen Comp Endocrinol 167: 35–43 doi: 10.1016/j.ygcen.2010.03.001 20211182 10.1016/j.ygcen.2010.03.001

[9] Odland GF. Skin in Histology (Greep R.O., ed.), 2^nd^ Edition McGraw-Hill Book Co; 1966 pp.420–445

[10] Flower DR (1996) The lipocalin protein family: structure and function. Biochem J 318: 1–14 8761444 10.1042/bj3180001 PMC1217580

[11] Brennan PA, Keverne EB (2004) Something in the air? New insights into mammalian pheromones. Curr Biol 14: 81–89 14738757 10.1016/j.cub.2003.12.052

[12] Beynon RJ, Hurst JL (2004) Urinary proteins and the modulation of chemical scents in mice and rats. Peptides 25: 1553–1563 doi: 10.1016/j.peptides.2003.12.025 15374657 10.1016/j.peptides.2003.12.025

[13] Lakshmi B, Mishra M, Srinivasan N, Archunan G (2015) Structure-based phylogenetic analysis of the lipocalin superfamily. PLoS One 10: e0135507 doi: 10.1371/journal.pone.0135507 26263546 10.1371/journal.pone.0135507 PMC4532494

[14] Pelosi P (1994) Odorant-binding proteins. Crit Rev Biochem Mol Biol 29: 199–228 doi: 10.3109/10409239409086801 8070277 10.3109/10409239409086801

[15] Cavaggioni A, Mucignat C, Tirindelli R (1999) Pheromone signalling in the mouse: Role of urinary proteins and vomeronasal organ. Arch Ital Biol 137: 193–200 10349497

[16] Cavaggioni A, Mucignat-Caretta C (2000) Major urinary proteins, alpha (2u)-globulins and aphrodisin. Biochim Biophys Acta 1482: 218–228 11058763 10.1016/s0167-4838(00)00149-7

[17] Hurst JL, Payne CE, Nevison CM, Marie AD, Humphries RE, Robertson DH, et al (2001) Individual recognition in mice mediated by major urinary proteins. Nature 414: 631–634 doi: 10.1038/414631a 11740558 10.1038/414631a

[18] Briand L, Trotier D, Pernollet JC (2004) Aphrodisin, an aphrodisiac lipocalin secreted in hamster vaginal secretions. Peptides 25: 1545–52 doi: 10.1016/j.peptides.2003.10.026 15374656 10.1016/j.peptides.2003.10.026

[19] Marchese S, Pes D, Scaloni A, Carbone V, Pelosi P (1998) Lipocalins of boar salivary glands binding odours and pheromones. Eur J Biochem 252: 563–568 9546674 10.1046/j.1432-1327.1998.2520563.x

[20] Zeng C, Spielman AI, Vowels BR, Leyden JJ, Biemann K, Preti G (1996) A human axillary odorant is carried by apolipoprotein D. Proc Natl Acad Sci USA 93: 6626–30 8692868 10.1073/pnas.93.13.6626 PMC39076

[21] D'Innocenzo B, Salzano AM, D'Ambrosio C, Gazzano A, Niccolini A, Sorce C, et al (2006) Secretory proteins as potential semiochemical carriers in the horse. Biochemistry 45: 13418–13428 doi: 10.1021/bi061409p 17087495 10.1021/bi061409p

[22] Rajkumar R, Karthikeyan K, Archunan G, Huang PH, Chen YW, Ng WV, et al (2010) Using mass spectrometry to detect buffalo salivary odorant-binding protein and its post-translational modifications. Rapid Commun Mass Spectrom 24: 3248–3254 doi: 10.1002/rcm.4766 20972998 10.1002/rcm.4766

[23] Robertson DHL, Beynon RJ, Evershed R (1993) Extraction, characterization and binding analysis of two pheromonally active ligands associated with major urinary protein of house mouse (*Mus musculus*). J Chem Ecol 19: 1405–1416 doi: 10.1007/BF00984885 24249171 10.1007/BF00984885

[24] Rajkumar R, Ilayaraja R, Liao CC, Archunan G, Achiraman S, Prakash S, et al (2010) Detection of alpha(2u)-globulin and its bound putative pheromones in the preputial gland of the Indian commensal rat (*Rattus rattus*) using mass spectrometry. Rapid Commun Mass Spectrom 24: 721–728 doi: 10.1002/rcm.4437 20169559 10.1002/rcm.4437

[25] Rajkumar R, Ilayaraja R, Mucignat C, Cavaggioni A, Archunan G (2009) Identification of alpha 2u-globulin and bound volatiles in the Indian common house rat (*Rattus rattus*). Indian J Biochem Biophys 46: 319–324 19788064

[26] Novotny MV (2003) Pheromones, binding proteins and receptor responses in rodents. Biochem Soc Trans 31: 117–122 doi: 10.1042/ 12546667 10.1042/bst0310117

[27] Ilayaraja R, Rajkumar R, Rajesh D, Muralidharan AR, Padmanabhan P, Archunan G (2014) Evaluating the binding efficiency of pheromone binding protein with its natural ligand using molecular docking and fluorescence analysis. Sci Rep 4: doi: 10.1038/srep05201 24903953 10.1038/srep05201 PMC4047529

[28] Laemmli UK (1970) Cleavage of structural proteins during the assembly of the head of bacteriophage T4. Nature 227: 680–685 5432063 10.1038/227680a0

[29] Rajkumar R, Prakash S, Archunan G, Sowdhamini R (2010) Primary structural documentation of the major urinary protein of the Indian commensal rat (*Rattus rattus*) using a proteomics platform. Protein Pept Lett 17: 449–457 20015025 10.2174/092986610790963573

[30] Atoji Y, Sugimura M, Suzuki Y (1989) Sebaceous glands of the infraorbital gland of the Japanese serow, *Capricornis crispus swinhoei*. J Submicrosc Cytol Pathol 21: 375–383

[31] Atoji Y, Yamamoto Y, Suzuki Y (1996) Infraorbital glands of a male Formosan serow (*Capricornis crispus swinhoei*). Eur J Morphol 34: 87–94 9090995 10.1076/ejom.34.2.87.13015

[32] Rajagopal T, Archunan G (2011) Histomorphology of preorbital gland in territorial and non-territorial male Blackbuck *Antelope cervicapra* L., a critically endangered species. Biologia 66: 370–378

[33] Richter J (1971) Unterschungen an antorbitaldrusen von Madaqua (Bovidae; Mammalia). Z Saugetierkunde 36: 334–342

[34] Cohen M, Gerneke DH (1976) Preliminary report on the intermandibular cutaneous glandular area and the infraorbital gland of the steenbok. J S Afr Vet Assoc 47: 35–37 1263192

[35] Mainoya JR (1978) Histological aspect of preorbital and interdigital glands of red duiker (*Cephalopus natalensis*). East Afr Wildl J 16: 256–272

[36] Sokolov VE, Chernova OF, Fekadu K (1994). The skin of some Ethiopian ungulates Print Ltd, Moscow. 194

[37] Kuhn HJ (1976) Antorbitaldruse und tranennasengang von Neotragus pygmaeus. Z Saugetierkunde 41: 369–380

[38] Kannan S, Ramesh Kumar K, Archunan G (1998) Sex attractants in male preputial gland: Chemical identification and their role in reproductive behavior of rats. Curr Sci 74: 689–691

[39] Fernald RD (2014) Communication about social status. Curr Opin Neurobiol 28: 1–4 doi: 10.1016/j.conb.2014.04.004 24793315 10.1016/j.conb.2014.04.004 PMC4177341

[40] Rajagopal T, Rajkumar R, Ponmanickam P, Achiraman S, Padmanabhan P, Archunan G (2015) Identification of pheromone-carrying protein in the preorbital gland post in the endangered Indian male Blackbuck *Antelope cervicapra* L. Indian J Exp Biol 53: 771–778 26742321

[41] Zhang JX, Sun L, Zhang JH, Feng Z (2008) Sex- and gonad-affecting scent compounds and 3 male pheromones in the rat. Chem Senses 33: 611–621 doi: 10.1093/chemse/bjn028 18515819 10.1093/chemse/bjn028 PMC2533420

[42] Agungpriyono S, Atoji Y, Yamamoto Y, Zuki AB, Novelina S (2006) Morphology of the intermandibular gland of the lesser mouse deer, *Tragulus javanicus*. Anat Histol Embryol 35: 325–333 doi: 10.1111/j.1439-0264.2006.00691.x 16968253 10.1111/j.1439-0264.2006.00691.x

[43] Achiraman S, Archunan G, Abirami B, Kokilavani P, Suriyakalaa U, SankarGanesh D, et al (2011) Increased squalene concentrations in the clitoral gland during the estrous cycle in rats: an estrus-indicating scent mark? Theriogenology 76: 1676–1683 doi: 10.1016/j.theriogenology.2011.06.033 21924481 10.1016/j.theriogenology.2011.06.033

[44] Stoddart DM (1980) The Ecology of Vertebrate Olfaction Chapman and Hall, London

[45] Pietras RJ (1981) Sex pheromone production by preputial gland: the regulatory role of estrogen. Chem Senses 4: 391–408

[46] Zhang JX, Rao XP, Sun L, Zhao CH, Qin XW (2007) Putative chemical signals about sex, individuality, and genetic background in the preputial gland and urine of the house mouse (*Mus musculus*). Chem Senses 32: 293–303 doi: 10.1093/chemse/bjl058 17251176 10.1093/chemse/bjl058

[47] Bacchini A, Gaetani E, Cavaggioni A (1992) Pheromone binding proteins of the mouse, *Mus musculus*. Experientia 48: 419–421 1374722 10.1007/BF01923448

[48] Dinh BL, Tremblay A, Dufour D (1965) Immunochemical study of rat urinary proteins, their relation to serum and kidney proteins (chromatographic separation of the major urinary protein). J Immunol 95: 574–582 4158465

[49] Briand L, Huet JC, Perez V, Lenoir G, Nespoulous C, Boucher Y, et al (2000) Odorant and pheromone binding by aphrodisin, a hamster aphrodisiac protein. FEBS Lett 476: 179–185 10913609 10.1016/s0014-5793(00)01719-1

[50] Stopkova R, Zdrahal Z, Ryba S, Sedo O, Sandera M, Stopka P (2010) Novel OBP genes similar to hamster Aphrodisin in the bank vole, *Myodes glareolus*. BMC Genomics 11: 45 doi: 10.1186/1471-2164-11-45 20085627 10.1186/1471-2164-11-45 PMC2824723

[51] Mastrogiacomo R, D’Ambrosio C, Niccolini A, Serra A, Gazzano A, et al (2014) An odorant-binding protein is abundantly expressed in the nose and in the seminal fluid of the rabbit. PLoS ONE 9(11): e111932 doi: 10.1371/journal.pone.0111932 25391153 10.1371/journal.pone.0111932 PMC4229146

[52] Armstrong SD, Robertson DH, Cheetham SA, Hurst JL, Beynon R (2005) Structural and functional differences in isoforms of mouse major urinary proteins: a male-specific protein that preferentially binds a male pheromone. Biochem J 391: 343–350 doi: 10.1042/BJ20050404 15934926 10.1042/BJ20050404 PMC1276933

